# Good concordance of HPV detection between cervico-vaginal self-samples and general practitioner-collected samples using the Cobas 4800 HPV DNA test

**DOI:** 10.1186/s12879-018-3254-y

**Published:** 2018-07-27

**Authors:** Mette Tranberg, Jørgen Skov Jensen, Bodil Hammer Bech, Jan Blaakær, Hans Svanholm, Berit Andersen

**Affiliations:** 1 Department of Public Health Programmes, Randers Regional Hospital, Skovlyvej 15, 8930 Randers NØ, Denmark; 2 Department of Clinical Medicine, Aarhus University, Palle Juul-Jensens Boulevard 82, 8200 Aarhus N, Denmark; 3 Research Unit for Reproductive Microbiology, Statens Serum Institut, Artillerivej 5, 2300 Copenhagen S, Denmark; 4 Section for Epidemiology, Department of Public Health, Aarhus University, Bartholins Allé 2, 8000 Aarhus C, Denmark; 5 Department of Obstetrics and Gynecology, Odense University Hospital, Sdr. Boulevard 29, 5000 Odense C, Denmark; 6 Department of Clinical Medicine, University of Southern Denmark, J.B. Winslows Vej 25, 5000 Odense C, Denmark; 7 Department of Pathology, Randers Regional Hospital, Østervangsvej 48, 8930 Randers NØ, Denmark

**Keywords:** Self-sampling, Human papillomavirus testing, Cervical cancer screening, Acceptability

## Abstract

**Background:**

Studies comparing self-samples and clinician-collected samples for high-risk human papillomavirus (HPV) detection using clinically validated PCR-based HPV DNA assays are limited. We measured the concordance of HPV detection between home-based self-sampling and general practitioner (GP) sampling using the Cobas 4800 HPV DNA test and studied women’s accept of home-based self-sampling.

**Methods:**

Paired GP-collected samples and cervico-vaginal self-samples were obtained from 213 women aged 30–59 years diagnosed with ASC-US within the cervical cancer screening program. After undergoing cervical cytology at their GP, the women collected a self-sample with the Evalyn Brush at home and completed a questionnaire. Both samples were HPV-tested using the Cobas 4800 test. Histology results were available for those who tested HPV positive in GP-collected samples.

**Results:**

We observed good concordance for HPV detection between self-samples and GP-collected samples (κ: 0.70, 95% CI: 0.58–0.81). No underlying CIN2+ cases were missed by self-sampling. Women evaluated that self-sampling was easy (97.2%, 95% CI: 93.9–98.9%) and comfortable (94.8%, 95% CI: 90.9–97.4%).

**Conclusions:**

Home-based self-sampling using the Evalyn Brush and the Cobas 4800 test is an applicable and reliable alternative to GP-sampling.

## Background

DNA tests for HPV testing in cervical cancer screening have been developed due to the strong causal relationship between persistent cervical infection caused by high-risk human papillomavirus (HPV) types and cervical cancer and its pre-cancer lesions [1, 2].

Screening based on HPV testing is more sensitive in detecting cervical intraepithelial neoplasia of grade 3 (CIN3) and cancer than cytology-based screening [3, 4], and women with a negative HPV test have a lower 5-year risk of CIN3 and cancer than women with a negative cytology [5]. An advantage of HPV testing is that, unlike cytology, it enables women to self-sample cervico-vaginal material at home (HPV self-sampling), which may improve cervical cancer screening participation [6].

Studies comparing self-samples and clinician-collected samples for HPV detection show moderate to very good concordance in referral populations [7, 8], whereas one study conducted in a screening population reported a very high level of agreement [9]. Yet, studies on HPV concordance derive mainly from studies using Hybrid Capture II (HC2) or in-house Polymerase Chain Reaction (PCR)-based assays [7, 8], while only few studies have used established, clinically validated PCR-based HPV DNA assays [9, 10].

A systematic review demonstrated that self-sampling is a well-accepted screening method. The main reported concerns were pain, discomfort touching themselves, and uncertainty as to whether the sample was collected correctly [11]. However, in most studies assessing acceptability, the women obtained the samples in clinics after face-to-face oral information from healthcare professionals, which is not the set-up if self-sampling is to be implemented in a routine screening program where self-sampling is to be home-based [11].

### Aims

This study aimed to measure concordance in HPV detection between paired self-samples collected at home using the Evalyn Brush device and general practitioner (GP)-collected samples, when using the clinically validated Cobas 4800 HPV DNA test. Further, we wanted to measure women’s accept of home-based self-sampling.

## Methods

### Setting

This study was conducted in the Central Denmark Region (CDR), which covers 23% of the Danish population [12]. Like the rest of the country, this region has been covered by a nationwide, organized, free-of-charge cervical cancer screening program since the late 1990s [13, 14].

Currently, 23–49-year-old women are invited for cervical cancer screening every third year, 50–64-year-old women every fifth year. A liquid-based cervical cytology sample is taken at their GP (GP-collected sample). The primary screening method is microscopic examination of the cytology sample for 23–59-year-old women; a primary HPV-DNA check-out test for 60–64-year-old women [13].

In the CDR, the Department of Pathology at Randers Regional Hospital handles all cytology and HPV analyses. As per routine, GPs obtain the sample using a cervical brush and the brush head is placed in 10 ml SurePath medium (BD Diagnostics, Burlington, NC) and mailed to the Pathology Department for further processing and testing according to guidelines, which for women aged 30–59 is microscopic examination. Women with high-grade cytological lesions (threshold ASC-H or HSIL or, AGC, or AIS or malignant tumor cells) are referred directly for colposcopy. Women diagnosed with ASC-US undergo routine reflex HPV DNA triage testing using the cell pellet from 1 mL SurePath medium. ASC-US/HPV-positive women are referred for colposcopy within 3 months. ASCUS/HPV-negative women are referred back to the routine screening program. Women with LSIL are monitored by repeated cytology testing [13].

For HPV DNA analyses, the Cobas 4800 assay (Roche Diagnostic, Switzerland) is used. This test is a real-time PCR fully automated method separately detecting HPV16, HPV18, and 12 other high-risk HPV types (HPV 31, 33, 35, 39, 45, 51, 52, 56, 58, 59, 66, and 68) using the β-globin gene as an extraction and amplification control [15].

### Inclusion of participants

Paired GP-collected samples and cervico-vaginal self-samples were obtained from 30 to 59-year-old women diagnosed with low-grade cytological lesions (ASC-US) within the screening program. As per routine, GP-collected samples from these women are all analyzed by both microscopy and HPV, which is not the case for younger and older women, or women with other cytological diagnoses [13].

Between June 2015 and December 2016, eligible women were consecutively identified daily through patient lists provided by the Department of Pathology. They received written information about the study and then contacted the investigator for oral information if they wanted to participate. A written and signed informed consent form had to be mailed back to the investigator before inclusion.

The exclusion criteria were pregnancy, giving birth less than < 3 months previously, and collecting the self-sample after colposcopy.

### Self-sampling collection, storage, and analysis

The women were sent a self-sampling kit comprising a brush device (Evalyn**®** Brush, Rovers Medical Devices B.V., Oss, Netherlands), written and picture-based user instructions showing how to collect the cervico-vaginal sample using the device, a questionnaire, and a pre-addressed return envelope.

Upon arrival at the laboratory, the brush head was placed in 10 ml of SurePath medium (BD Diagnostics, Burlington, NC), stored overnight at 4 °C, and then vortexed for 5 min. A 6.4 ml volume of the self-sample material was centrifuged at 3000 x RPM for 20 min at room temperature. After centrifugation, with supernatant removed, the cell pellet was placed in 1 mL 25% ethanol-buffered (TRIS) and stored at -80 °C, until further processing. A volume of 6.4 ml was chosen to adjust for the material volume used for cytology examination performed on the GP-collected samples.

The self-samples were thawed overnight at 4 °C before the day of analysis. For analysis, the self-sample (1 ml volume) was vortexed for 15 s before being placed in test tubes, being the starting point for the HPV testing. Each run included four water samples to measure contamination. All HPV testing was performed following the manufacturers’ instructions using a protocol without a sample pre-heating step which was also the case for the GP-collected samples. The investigator and the laboratory personnel performing the HPV testing were blinded to the HPV results of the GP-collected samples.

### Questionnaire

The questionnaire included three questions on self-sampling experience and one on the clarity of the user instructions. To avoid low frequencies, the responses were grouped into three groups “Agree” (totally agree or agree), “Disagree” (disagree or totally disagree), and “Do not know”. Multiple response answers were not allowed. The women were asked to record the date they had taken the self-sample, whether they had had sexual intercourse in between the two samplings, and their partner status (i.e., regular partner). Open feedback was possible. The data were double-entered into REDCap [16].

### Data on test results

Information on the HPV self-sample test results was obtained through patient lists provided by the Department of Pathology. Data on the GP-collected HPV test results and the histological results were retrieved from the nationwide Danish Pathology Data Bank (DPDB), which has been complete since the late 2000s [17]. Histological results were available only for women who had tested HPV-positive in their GP-collected sample and were classified using the CIN classification, which was grouped into normal, CIN, CIN1, and CIN2+ (including CIN2, CIN3/AIS, and carcinoma). To ascertain the histological result, the most severe diagnosis was used if more were available.

### Sample size

The study was designed to estimate a 95% confidence interval (CI) with a width of +/− 5%. With an expected 86% sensitivity and 85% specificity of HPV detection (high-risk HPV types) using the Evalyn Brush and a PCR-based HPV DNA test, a minimum of 198 women had to be included [10].

### Statistical analyses

The HPV concordance between the paired samples was assessed using the Kappa statistic (Cohen’s Kappa, κ) and defined as “Poor” (κ ≤ 0.20), “Fair” (0.21 ≤ κ ≤ 0.40), “Moderate” (0.41 ≤ κ ≤ 0.60), “Good” (0.61 ≤ κ ≤ 0.80), or “Very good” (κ ≥ 0.81) [18]. The overall percentage of agreement between the paired samples was calculated as the proportion of concordant sample sets divided by the total number of samples. We calculated the sensitivity and specificity of HPV detection in the self-samples with corresponding 95% CIs based on the binomial distribution using the GP-collected samples as reference standard. When assessing the HPV concordance regarding specific genotypes (HPV16/18 and HPV other), the genotypes were defined as “HPV16/18” (HPV16 and/or HPV18 including those having co-infections with other HPV types) and “HPV other” (HPV of other types including those having co-infections with HPV16/18). Concordance was determined as at least one identical genotype in both samples; discordance was determined as no genotype similarities. For continuous data, medians and interquartile ranges (IQR) were calculated; the Mann Whitney rank sum test was used to test for differences. Descriptive statistics (proportions and 95% CIs) were used to measure the women’s accept of self-sampling. The χ2-test was used to test for differences in categorical data.

*P*-values < 0.05 were considered statistically significant. All statistical analyses were performed using STATA, version 14 (STATA College).

### Ethical approval

The study was approved by the local Ethical Committee of the Central Denmark Region (journal no.: 1–16–02-209-15) and by the Danish Data Protection Agency (journal no.:1–10–72-69-15).

## Results

### Participant characteristics

From a total of 1110 eligible women, 216 (19.5%) returned a self-sample. Three women (0.3%) were excluded because the self-sample was taken after the biopsy, leaving 213 (19.2%) women for analysis.

The included women’s median age was 44 years (IQR: 38–49 years). The majority were aged 40 to 49 years (*n* = 113, 53.0%) followed by women aged 30 to 39 years (*n* = 59, 27.7%) and 50 to 59 years (*n* = 41, 19.3%). All paired GP-collected samples and self-samples were valid for HPV testing.

The median number of days between the GP-collected sample and the self-sample was 43 days (IQR: 34–53 days, range: 13–95 days). Histological results were available for 46 women, of whom 19 (41.3%) had a normal biopsy, 4 had CIN (not specified) (8.7%), 11 had CIN1 (23.9%), and 12 (26.1%) had CIN2+ (2 CIN2, 9 CIN3, 1 adenocarcinoma).

### HPV prevalence and concordance between self-sampling and GP-sampling

For self-samples, the HPV prevalence (any type) was 24.4% (95% CI: 18.8–30.8%); for GP-collected samples 22.1% (95% CI: 16.7–28.2%) (Table 1). There was good concordance for HPV detection between the self-samples and the GP-collected samples (κ: 0.70, 95% CI: 0.58–0.81). The overall level of agreement was 89.2% (95% CI: 84.2–93.0%) (Table 1). For HPV detection in self-samples, the sensitivity was 80.9% (95% CI: 66.7–90.9%) and the specificity 91.6% (95% CI: 86.3–95.3%) when the GP-collected sample was used as reference.

**Table 1:** Concordance and agreement for HPV detection (any type) between self-samples and GP-collected samples

	GP-collected samples						κ^b^ (95% CI)	Agreement (%) (95% CI)	Sensitivity (%) (95% CI)	Specificity (%) (95% CI)
	HPV any positive^a^		HPV negative		Total					
	n	%	n	%	n	%				
Self-samples										
HPV any positive^a^	38	17.8	14	6.6	52	24.4	0.70 (0.58–0.81)	89.2 (84.2–93.0)	80.9 (66.7–90.9)	91.6 (86.3–95.3)
HPV negative	9	4.2	152	71.4	161	75.6				
Total	47	22.1	166	77.9	213	100.0				

^a^HPV any positive: HPV16 and/or HPV18 and/or HPV31, 33, 35, 39, 45, 51, 52, 56, 58, 59, 66, and 68. % = Row percentage

^b^Cohen’s Kappa. “Poor” (κ ≤ 0.20), “Fair” (0.21 ≤ κ ≤ 0.40), “Moderate” (0.41 ≤ κ ≤ 0.60), “Good” (0.61 ≤ κ ≤ 0.80), or “Very good” (κ ≥ 0.81) (18)

A total of 23 women (10.7%) had disconcordant results. In nine cases, the women were GP-collected sample HPV-positive/self-sample HPV-negative. For these women, the median number of days between the two samples was insignificantly higher than in women with concordant sample results (*n* = 38) (40 versus 35 days, respectively) (*p* = 0.20). None of these nine women reported having trouble taking the self-sample, although one woman responded feeling insecure if she had collected the self-sample correctly. Histological results were available for eight out of the nine women, of whom five (62.5%) had no CIN, one (12.5%) had CIN (not specified), and two women (25.0%) had CIN1. The 12 women diagnosed with CIN2+ all had HPV detected in their self-sample.

Fourteen women were GP-collected sample HPV-negative/self-sample HPV-positive; two of whom (14.2%) reported no sexual intercourse in the time span separating the two samples.

The HPV prevalence was insignificantly higher in self-samples than in GP-collected samples for HPV16/18 (9.4%, 95% CI: 5.8–14.1%) versus (8.0%, 95% CI: 4.7–12.5%) and HPV of other types (21.1%, 95% CI: 15.8–27.2) versus (18.3%, 95% CI: 13.4–24.2%) (Table 2). Concordance for HPV16/HPV18 detection between self-samples and the GP-collected samples was good (κ: 0.73, 95% CI: 0.57–0.90) with overall 95.7% agreement (95% CI: 92.1–98.0%) (Table 2). For HPV16/18, the sensitivity and specificity in self-samples as compared with GP-collected samples was 82.4% (95% CI: 56.7–96.2%) and 96.9% (95% CI: 93.5–98.8%), respectively. For HPV of other types, a good concordance was seen between self-samples and GP-collected samples (k = 0.64, 95% CI: 0.51–0.78), with an overall agreement of 88.7% (95% CI: 83.7–92.6%) (Table 2). The corresponding sensitivity was 76.9% (95% CI: 60.7–88.9%); the specificity 91.4% (95% CI: 86.2–95.1%).

**Table 2:** Concordance and agreement between self-samples and GP-collected samples according to specific genotypes

	GP-collected samples						κ^a^ (95% CI)	Agreement (%) (95% CI)	Sensitivity (%) (95% CI)	Specificity (%) (95% CI)
	HPV 16/18^b^ positive		HPV16/18^b^ negative		Total					
	n*	%	n*	%	n	%				
Self-samples										
HPV 16/18^b^ positive	14	6.6	6	2.8	20	9.4	0.73 (0.57–0.90)	95.7 (92.1–98.0)	82.4 (56.7–96.2%)	96.9 (93.5–98.8%)
HPV 16/18^b^ negative	3	1.4	190	89.2	193	90.6				
Total	17	8.0	196	92.0	213	100				
	HPV other^c^ positive		HPV other^c^ negative		Total					
Self-samples	n*	%	n*	%	n					
HPV other^c^ positive	30	14.1	15	7.0	45	21.1	0.64 (0.51–0.78)	88.7 (83.7–92.6)	76.9 (60.7–88.9%)	91.4 (86.2–95.1%)
HPV other^c^ negative	9	4.2	159	74.6	168	78.9				
Total	39	18.3	174	81.7	213	100				

n* = Women with co-infections with HPV16/18 and HPV of other types appear in both sub-analyses

^a^Cohen’s Kappa. “Poor” (κ ≤ 0.20), “Fair” (0.21 ≤ κ ≤ 0.40), “Moderate” (0.41 ≤ κ ≤ 0.60), “Good” (0.61 ≤ κ ≤ 0.80), or “Very good” (κ ≥ 0.81) (18)

^b^HPV16/18: HPV16 and/or HPV18 including co-infections with HPV of other types (HPV31, 33, 35, 39, 45, 51, 52, 56, 58, 59, 66, and 68)

^c^HPV other: HPV31, 33, 35, 39, 45, 51, 52, 56, 58, 59, 66, and 68 including co-infections with HPV16/18. % = Row percentage

### Women’s accept of self-sampling

**Fig. 1:**
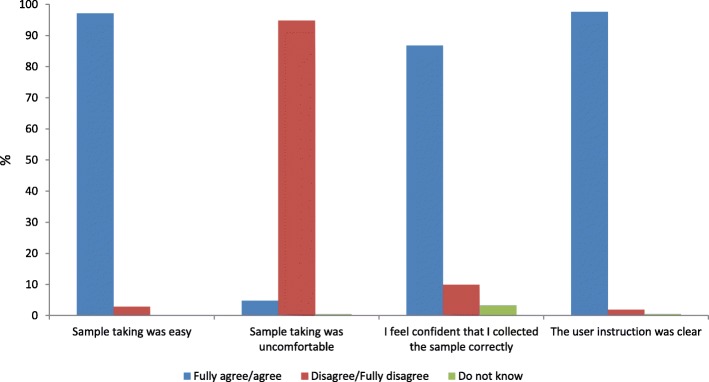
The women’s accept of self-sampling

A total of 212 out of 213 women answered the questionnaire. Self-sampling with the Evalyn Brush was recorded as easy by 97.2% (95% CI: 93.9–98.9%) (Fig. 1), while 6 (2.8%, 95% CI: 1.0–6.1%) recorded that self-sampling was not easy. Most women reported that self-sampling was comfortable (94.8%, 95% CI: 90.9%–97.4%). A total of 21 (9.9, 95% CI: 6.2–14.7%) women reported uncertainty about performing the self-sample correctly, with three women reporting that they felt insecure because it was difficult to hear the click when rotating the brush. In 86.8% (95% CI: 81.5–91.0%) of the cases, the women felt confident that they collected the self-sample correctly. Most women responded that the user instructions were clear (97.6%, 95% CI: 94.6–99.2%). There were no statistically differences between the age groups (*p* = 0.33) for any of the questions.

## Discussion

### Main findings

Our study showed good concordance in HPV detection between paired self-samples and GP-collected samples. Home-based self-sampling using the Evalyn Brush was a well-accepted screening method. Compared with GP-sampling, no cases of underlying CIN2+ were overlooked by self-sampling.

### Strengths and limitations

A key strength of our study was that we used a combination of a clinically validated self-sampling device and a clinically validated automated PCR-based HPV DNA test assay on paired samples [10, 19]. Furthermore, women collected the self-sample at home without supervision by healthcare professionals, which is the most relevant setting for testing self-sampling before its rollout in a routine screening program.

The main limitation is the time span between the GP-collected samples and self-samples, making the results not directly comparable. Still, the questionnaire data helped us to interpret discordant results. Another explanation for the discordant results could be that the self-samples had been subjected to freezing at -80 °C prior to the HPV testing which potentially could have affected the amount of HPV DNA in the self-samples, unlike the GP-collected samples which have not been frozen. However, since DNA is generally considered to be stable at -80 °C, we assume that this has not significantly affected the results and conclusions of this study. Our study population comprised women with ASC-US of whom one fourth was referred for colposcopy due to concurrent HPV infection; thus, histological results were not available for women with HPV-negative GP-collected samples. Ideally, histological results should have been available from all women. This was not possible in our set-up. Still, the available histological results allowed us to make the important conclusion that no cases of underlying CIN2+ had been overlooked by self-sampling which is important when implementing self-sampling in routine screening practice.

Even though our study population can be considered a “low-risk” population compared with the referral populations that have typically been targeted in similar studies [7], our population is still not representative of a screening population. Consequently, this study cannot be generalized to such populations.

### Interpretation and comparison with previous studies

The concordance in our study (k = 0.70) was comparable with the mean k (k = 0.71) reported for brush devices combined with PCR-based HPV DNA tests in the review by Schmeink et al. [7], but higher than the mean k (k = 0.66) in the review by Petiginat et al. [8]. This difference might be explained by differences in self-sampling devices, HPV tests, laboratory protocols, and study populations (screening or referral population). In our study, some of the discrepancy in the HPV concordance between self-sampling and GP-sampling could plausibly also be explained by spontaneous clearance or a new HPV exposure due to the time span separating the samples.

In our study, the sensitivity and specificity of HPV detection (of any type) was 80.9% and 91.6% in self-samples, respectively, when using the GP-collected samples as reference standard. These results are comparable to those by Van Baars et al. [10] who found a sensitivity of 82.7% and a specificity of 89.5% for HPV detection using the Evalyn Brush together with the clinically validated PCR-based GP5+/6+ HPV DNA test in a referral population. Thus, self-sampling using validated HPV DNA analyses seems feasible, but the optimal combination of self-sample device and HPV DNA test remains unknown.

Ketelaars et al. [9] found no significant differences in HPV16/18 prevalence between samples, whereas the prevalence of HPV of other types was significantly higher in self-samples (8.0%) than in GP-collected samples (6.3%). We observed no significant differences in the HPV prevalence between samples, but the same trend was seen, especially for the prevalence of HPV of other types (21.1% versus 18.3%, respectively). The higher HPV prevalence in self-samples compared with GP-collected samples increases referral rates, especially because reflex cytology triage is not possible on self-sampled material. To avoid excessive referral rates in women with HPV-positive self-samples without underlying CIN2+, a direct triage method like DNA methylation [20] could be considered to reduce referral rates and prevent overtreatment.

Most importantly, we showed that no underlying CIN2+ cases were overlooked by self-sampling. Some of the women in the GP-collected HPV negative/self-sample HPV-positive group might possibly have had underlying CIN2+, but this cannot be explored further in this study since referral for colposcopy was based on the result of the GP-collected sample.

The self-sampling device and user instructions must be acceptable if we wish to improve screening participation by this method. In our study, more than 85% expressed confidence in having collected the self-sample correctly, and only 5% expressed discomfort with collecting the sample. Hence, home-based self-sampling using the Evalyn Brush appeared to be a well-accepted screening method. Yet, almost 10% reported uncertainty regarding sample collection and some stated the lack of a click when rotating the brush as their reason. Despite small numbers, this finding is higher than reported by Van Baars et al. [10] (3.0%) and Ketelaars et al. [9] (0.8%) who used the same device in a referral and screening population, respectively. This difference might be explained by the fact that the women in our study performed home-based self-sampling, whereas in the study by Van Baars et al. [10] the women performed clinic-based self-sampling allowing them to ask questions and receive guidance. Nevertheless, our results suggest that if home-based self-sampling were to be rolled out in a routine setting, it should be considered giving women the opportunity to contact healthcare professionals for guidance.

## Conclusions

We report a high acceptability of home-based self-sampling and good concordance between self-samples and GP-collected samples in terms of HPV detection using the clinically validated Cobas 4800 HPV DNA test. Our findings demonstrate the feasibility of offering self-sampling as an alternative to GP-sampling in cervical cancer screening.

## Abbreviations

AGC: Atypical Glandular Cells
AIS: Adenocarcinoma In Situ
ASC-H: Atypical Squamous Cells cannot exclude HSIL
ASC-US: Atypical Squamous Cells of Undetermined Significance
CDR: Central Denmark Region
CI: Confidence interval
CIN: Cervical Intraepithelial Neoplasia
CIN1: Cervical Intraepithelial Neoplasia of grade 1
CIN2 +: Cervical Intraepithelial Neoplasia of grade 2 or worse
CIN3: Cervical Intraepithelial Neoplasia of grade 3
DPDB: Danish Pathology Data Bank
GP: General Practitioner
HC2: Hybrid Capture II
HPV: Human Papilloma Virus
HSIL: High-grade Squamous Intraepithelial Lesion
IQR: InterQuartile Ranges
LSIL: Low-grade Squamous Intraepithelial Lesion
PCR: Polymerase Chain Reaction
